# Effectiveness of a Technology-Based Supportive Educational Parenting Program on Parental Outcomes (Part 1): Randomized Controlled Trial

**DOI:** 10.2196/10816

**Published:** 2019-02-13

**Authors:** Shefaly Shorey, Yvonne Peng Mei Ng, Esperanza Debby Ng, An Ling Siew, Evalotte Mörelius, Joanne Yoong, Mihir Gandhi

**Affiliations:** 1 Clinical Research Centre Alice Lee Centre for Nursing Studies National University of Singapore Singapore Singapore; 2 National University Hospital Singapore Singapore; 3 National University of Singapore Singapore Singapore; 4 Linkoping University Linkoping Sweden; 5 Singapore Clinical Research Institute Singapore Singapore

**Keywords:** parents, social support

## Abstract

**Background:**

Transitioning into parenthood can be stressful for new parents, especially with the lack of continuity of care from health care professionals during the postpartum period. Short hospital stays limit the availability of support and time parents need to be well equipped with parenting and infant care skills. Poor parental adjustment may, in turn, lead to negative parental outcomes and adversely affect the child’s development. For the family’s future well-being, and to facilitate a smoother transition into parenthood, there is a need for easily accessible, technology-based educational programs to support parents during the crucial perinatal period.

**Objective:**

This study aimed to examine the effectiveness of a technology-based supportive educational parenting program (SEPP) on parenting outcomes during the perinatal period in couples.

**Methods:**

A randomized, single-blinded, parallel-armed, controlled trial was conducted. The study recruited 236 parents (118 couples) from an antenatal clinic of a tertiary hospital in Singapore. Eligible parents were randomly assigned to the intervention group (n=118) or the control group (n=118). The SEPP is based on Bandura’s self-efficacy theory and Bowlby’s theory of attachment. Components of the intervention include 2 telephone-based educational sessions (1 antenatal and 1 immediately postnatal) and a mobile health app follow-up for 1 month. The control group only received routine perinatal care provided by the hospital. Outcome measures including parenting self-efficacy (PSE), parental bonding, perceived social support, parenting satisfaction, postnatal depression (PND), and anxiety were measured using reliable and valid instruments. Data were collected over 6 months at 4 time points: during pregnancy (third trimester), 2 days postpartum, 1 month postpartum, and 3 months postpartum. Outcomes were standardized using baseline means and SDs. Linear mixed models were used to compare the groups for postpartum changes in the outcome variables.

**Results:**

The intervention group showed significantly better outcome scores than the control group from baseline to 3 months postpartum for PSE (mean difference, MD, 0.37; 95% CI 0.06 to 0.68; *P=*.02), parental bonding (MD −1.32; 95% CI −1.89 to −0.75; *P*<.001), self-perceived social support (MD 0.69; 95% CI 0.18 to 1.19; *P=*.01), parenting satisfaction (MD 1.40; 95% CI 0.86 to 1.93; *P*<.001), and PND (MD −0.91; 95% CI −1.34 to −0.49; *P*<.001). Postnatal anxiety (PNA) scores of the intervention group were only significantly better after adjusting for covariates (MD −0.82; 95% CI −1.15 to −0.49; *P*<.001).

**Conclusions:**

The technology-based SEPP is effective in enhancing parental bonding, PSE, perceived social support and parental satisfaction, and in reducing PND and PNA. Health care professionals could incorporate it with existing hands-on infant care classes and routine care to better meet parents’ needs and create positive childbirth experiences, which may in turn encourage parents to have more children.

**Trial Registration:**

ISRCTN Registry ISRCTN48536064; http://www.isrctn.com/ISRCTN48536064 (Archived by WebCite at http://www.webcitation.org/6wMuEysiO).

## Introduction

### Background

Singapore’s fertility rate has been declining over the years despite the government’s desperate attempts to incentivize married couples to have more children. Career prioritization [1], previous negative childbirth experiences, and unmet parental expectations were the main reasons for this declining trend [2,3]. To mitigate negative childbirth experiences and prepare parents for parenthood, perinatal educational classes have been made available in Singapore’s hospitals. However, owing to unawareness, time, and financial constraints, only few parents attend these classes [4]. The dissemination of overwhelming infant care information through pamphlets or in a didactic style during the short hospital stay also tends to cause information overload for parents [4-6]. Despite the growing interest and involvement of fathers in parenting [5], perinatal care by Singapore’s hospitals still focuses primarily on breastfeeding and the physical health of the mother and child, failing to consider paternal involvement and the importance of parent-child bonding [5,7]. Recently, under a smart nation initiative, Singapore aimed to deliver holistic health care through technological innovations [8]. Given the increasing number of parents relying on Web-based information and online support communities [9,10], and considering the unreliability of sources and lack of professional moderation of Web-based information [11], there is a need for an improved technology and a theory-based perinatal educational program for parents.

Among all parental outcomes, parenting self-efficacy (PSE) is a major determinant of a positive parenting experience. According to Bandura, self-efficacy refers to one’s feeling of effectiveness in accomplishing required tasks and activities [12]. For better PSE, Bandura emphasized that parents must have confidence in their ability to perform specific skills and believe that their actions will have the desired outcomes to ensure successful parenting [13]. Self-efficacy can be developed through the mastery of experiences, vicarious experiences, social persuasion, and affective and physiological factors [12]. Especially for first-time parents, PSE is highly associated with better coping responses and parenthood adjustment and positive psychological and developmental outcomes for parents and children [14,15]. In addition, Bowlby’s attachment theory [16] also theorizes that PSE, social support, and parental emotional well-being are essential to establishing early parent-infant bonding, which is the foundation of a positive development of social relationships in infants. Therefore, it is important to investigate the relationships among PSE, social support, parent-infant bonding, parents’ psychological health (ie, postnatal depression [PND] and anxiety), and parenting satisfaction.

During the postpartum period, the stressful adaptation to new parental roles, additional infant care responsibilities, and a lack of social support from one’s partner [17-19] can adversely affect parental bonding with the infant, which may give rise to child developmental and attachment issues [20]. Poor parental adjustment also increases the risks of postpartum psychological disorders in both mothers and fathers [21,22]. On the contrary, high levels of perceived PSE [23] and social support help to facilitate smoother transition to parenthood [24], leading to increased parental bonding, parenting satisfaction, and parenting competence [25], and lowered risks of PND [23] and postnatal anxiety (PNA) [26]. This is evident in the vital role of PSE in promoting a positive parenting experience. Hence, intervention programs should emphasize on increasing PSE among parents.

In a recent review, educational intervention for parents was shown to be effective in enhancing and sustaining PSE long-term [27]. This corresponds with previous technology-based intervention studies, which were shown to not only boost PSE [28-31] but also increase parenting satisfaction [28,30,31], parental bonding [32,33], and perceived social support [29,31,34-36] and reduce postpartum psychological disorders [32,35,37]. However, most of these interventions cater only to mothers [32,35,37,38] and are only introduced during the postnatal period [28,30,31,33,34,38]. This study addresses the lack of continuity of care during the perinatal period [4,39] and the lack of inclusion of fathers’ involvement in parenting [28,31] by providing new insights with a technology- and couple-based parenting educational program made available during the perinatal period.

### Aim and Hypotheses

This study aimed to examine the effectiveness of a technology-based supportive educational parenting program (SEPP) on parental outcomes in terms of PSE, parental bonding, perceived social support, parenting satisfaction, PND, and PNA during the perinatal period in couples.

Compared with the control group receiving standard care, we hypothesized that the intervention group will have significantly better scores for PSE, perceived social support, parental bonding, and parental satisfaction, and lower scores for PND and PNA from baseline to 3 months postpartum.

## Methods

### Study Design

This was a randomized, single-blinded, parallel-armed controlled trial. The research assistant responsible for data collection was blinded to the intervention assignment of participants. Before recruitment, an independent statistician generated a randomization list using a permuted block randomization method (no stratification factor) with a 1:1 allocation ratio using Research Randomizer [40]. The block size was blinded to the study team. Couples were randomized into 2 groups (59 couples in each group) using an opaque envelope containing nonduplicated numbers (1-118).

### Eligibility Criteria

Eligible participants were heterosexual married couples aged 21 years and older (individuals aged 21 years and below are considered minors in Singapore), were proficient in spoken and written English, owned a mobile phone with internet access, and planned to stay in Singapore for the first 3 months post delivery. Only mothers who had a low-risk singleton pregnancy with more than 28 weeks gestation were included. Fathers and mothers were excluded if they were a single parent, had self-reported physical or mental disorders that would interfere with their ability to participate in the study “and” or “or” if the mother had a high-risk pregnancy (eg, placenta-previa major, preeclampsia, or pregnancy-induced hypertension), had assisted delivery such as vacuum or forceps with a fourth degree perineal tear, and/or had given birth to a stillborn or newborn with congenital abnormalities and/or medical complications. Mothers with high-risk pregnancy were excluded to reduce confounding influences on the parental outcome scores. Upon recruitment, couples were informed of possible exclusion from the study if mothers were to experience complications during pregnancy and/or delivery.

### Intervention

Parents assigned to the control group received routine perinatal care provided by the hospital, which includes antenatal checkups with an obstetrician, optional antenatal educational classes and postnatal parent-craft educational classes, and regular follow-ups with doctors from 10 days to 6 weeks postpartum.

Parents in the intervention group received the SEPP in addition to the standard routine perinatal hospital care. The SEPP adopted a 3-step approach, including (1) a 30-min telephone-based antenatal educational session, (2) a 60-min telephone-based immediate postnatal educational session, and (3) a mobile health (mHealth) app follow-up educational session made available for 4 weeks postpartum. Individual usernames, masking the parents’ identities, and passwords were issued to the parents for access to the mHealth app. Details of the SEPP are summarized in the protocol [41].

The mHealth app contained knowledge-based content that addressed issues on breastfeeding, maternal self-care, newborn care tasks, dealing with emotional challenges, and enhancing parental efficacy and bonding, besides providing insights to new parents to facilitate their transition into parenthood. In addition, parental queries could be posted in the app’s discussion forum, which were answered daily by a trained midwife for the first 4 weeks post childbirth. Parents were also highly encouraged to share their personal insights and experiences in response to such queries. The mHealth app issued daily push notifications regarding important milestones on parenting. Further specifications on the intervention can be found in the study protocol [41].

### Procedure

The study took place in the antenatal clinic of a tertiary hospital, National University Hospital, in Singapore from December 2016 to December 2017. Participants were recruited as a couple (father and mother dyad) when they went for their routine antenatal checkup at the antenatal clinic. With the support and referral of nurse managers and clinicians at the antenatal clinic, a research assistant (RA1) approached referred couples to explain the purpose and details of the study. After being screened for eligibility, interested couples gave their informed consent and had to complete a demographics form and baseline questionnaire. They were then randomized into either the intervention group or the control group. For the SEPP intervention group, the RA1 proceeded to deliver a 30-min telephone-based antenatal educational session to participants. After childbirth, the couples were reapproached by the RA1 in the postnatal wards to finish another set of questionnaires. They then received a 60-min telephone-based postnatal educational session conducted by the RA1. Before discharge from the hospital, the couples were required to download the supportive parenting educational mHealth app. The RA1 guided couples through the app’s functions on the spot. Individual usernames and passwords, which would expire in 4 weeks, were provided for access to the mHealth app.

For couples in the control group, only routine perinatal care by the hospital was provided. Subsequent postbaseline data collection was done through telephone calls by another research assistant (RA2) who was blinded to the group allocation. Data collection took place at the following time points for all parents: (1) during pregnancy (baseline), (2) 2 days postpartum (immediate), (3) 4 weeks (1 month) postpartum, and (4) 3 months postpartum.

### Outcome Measures

The primary outcome (PSE) and secondary outcomes (parental bonding, PND, PNA, perceived social support, and parenting satisfaction) were measured using validated and reliable self-report questionnaires. PSE was measured using the 10-item Parenting Efficacy Scale (PES) [42], with a score range of 10 to 40. A high PES score indicates a high level of perceived self-efficacy [43]. Internal consistency of the PES was high across all time points (baseline, immediate postpartum, 1 month postpartum, and 3 months postpartum) with standardized Cronbach alphas of .935, .928, .925, and .868, respectively. Parental bonding was measured using the 8-item Parent-to-Infant Bonding Questionnaire (PIBQ) [44], which has a 4-point Likert scale. As the eighth item on aggression toward newborns was found to be poorly correlated with other items, this item was dropped to improve internal consistency among items and to help in calculating the total score. Total scores range from 0 to 21, and a score of 2 and above for each item suggests poor and ineffective parental bonding [30,45,46]. Standardized Cronbach alphas for the PIBQ were .704, .585, .663, and .624 across each time point. The 10-item Edinburgh Postnatal Depression Scale (EPDS), with a score range of 0 to 30, was used to measure PND [47]. A higher score indicates a higher risk of PND. The recommended cutoff score for PND screening in mothers is 12 or 13 [48], whereas the recommended cutoff score for PND in fathers is above 10 [49]. The EPDS had high internal consistency across each time point (.811, .834, .853, and .834). The 40-item State Trait Anxiety Inventory (STAI) [50] is widely used to measure PNA and has a score range of 40 to 160, with a higher score indicating a higher level of parental anxiety [31,51]. The internal consistencies of STAI were .957, .962, .964, and .961 across each time point. The 8-item Perceived Social Support for Parenting (PSSP) [52] scale constitutes 2 subparts (4 items each) that are used to measure parents’ perceived social support received from their partner and others. It has a total score range of 0 to 40, with a higher score indicating higher perceived social support [52] *.* The internal consistencies of the PSSP scale were .936, .875, .923, and .936 across each time point. Finally, parenting satisfaction was measured with an evaluation subscale of the What Being a Parent of a Baby Is Like (WPBL) scale [46]. It consists of 11 items, each with a 10-point semantic differential scale ranging from 0 to 9. A higher score indicates higher parenting satisfaction [53,54]. The WPBL scale has high internal consistencies of .956, .929, .958, and .954 across each time point. Further details on the psychometric properties of each outcome measure are mentioned in the protocol paper [41].

### Data Analysis

The sample size was calculated based on a repeated measure analysis accounting for intracluster (within couple) to examine the differences between the 2 groups (intervention and control) at 3 months. Assuming a medium effect size of 0.3, an intracluster correlation of .05, and a correlation between repeated measurements of .5 at a power of 85% with a significance level of 5% (2-sided), 88 participants (44 couples) in each group were required. Factoring a 25% attrition rate, 236 participants (118 couples), with 118 participants (59 couples) in each group, were required.

The analysis was performed on the intention-to-treat population. All outcome data were treated continuously. As the total scores for the outcome variables (PSE, PIBQ, EPDS, STAI, PSSP, and WPBL) were on different scales, they were standardized to a z-score using their baseline mean and SD. This will enable interpreting all the outcome variables on the same scale, equivalent to the standardized effect size. In this study, each couple should be considered as a cluster of 2 individuals whose outcomes could be correlated. To account for intracluster correlation within clusters, linear mixed-effect models were used to compare the 2 groups on change in PSE, PIBQ, EPDS, STAI, PSSP, and WPBL z-scores at immediate, 1 month, and 3 months postpartum. The unadjusted model included couple-specific random intercepts, and baseline value, indicator variable for the intervention (reference—control), indicator variables for time points: immediate, 1 month, 3 months postpartum (reference—baseline), and interaction between indicator variables for intervention and 3 time points as fixed effects. A sensitivity analysis was performed using the same model adjusted for age, gender, ethnicity, education, employment status, household income, length of marriage, antenatal class attendance, confinement period, maternal/paternal leave, and mode of feeding. Similar models were performed separately for mothers and fathers to understand the intervention effect in each of these subgroups. Finally, the unadjusted and adjusted analyses were performed using the complete case data and multiple imputed data based on the Markov Chain Monte Carlo method (50 imputations) to assess the robustness of the model in the presence of missing data. A *P* value (*P*) of less than .05 was considered statistically significant. The analysis was performed using IBM SPSS 24.0.

### Ethical Considerations

This study received ethics approval from the National Health Group Domain Specific Review Board (Ref. No: NHG DSRB: 2016/00651) before recruitment of participants commenced. All participants were given a participant information sheet and were briefed thoroughly on the purpose of the study and procedures before their consent was obtained. Participation was strictly voluntary, and anonymity was guaranteed. Participants were also informed of the right to withdraw at any point of the study without consequences.

## Results

### Participants’ Details

**Figure 1:**
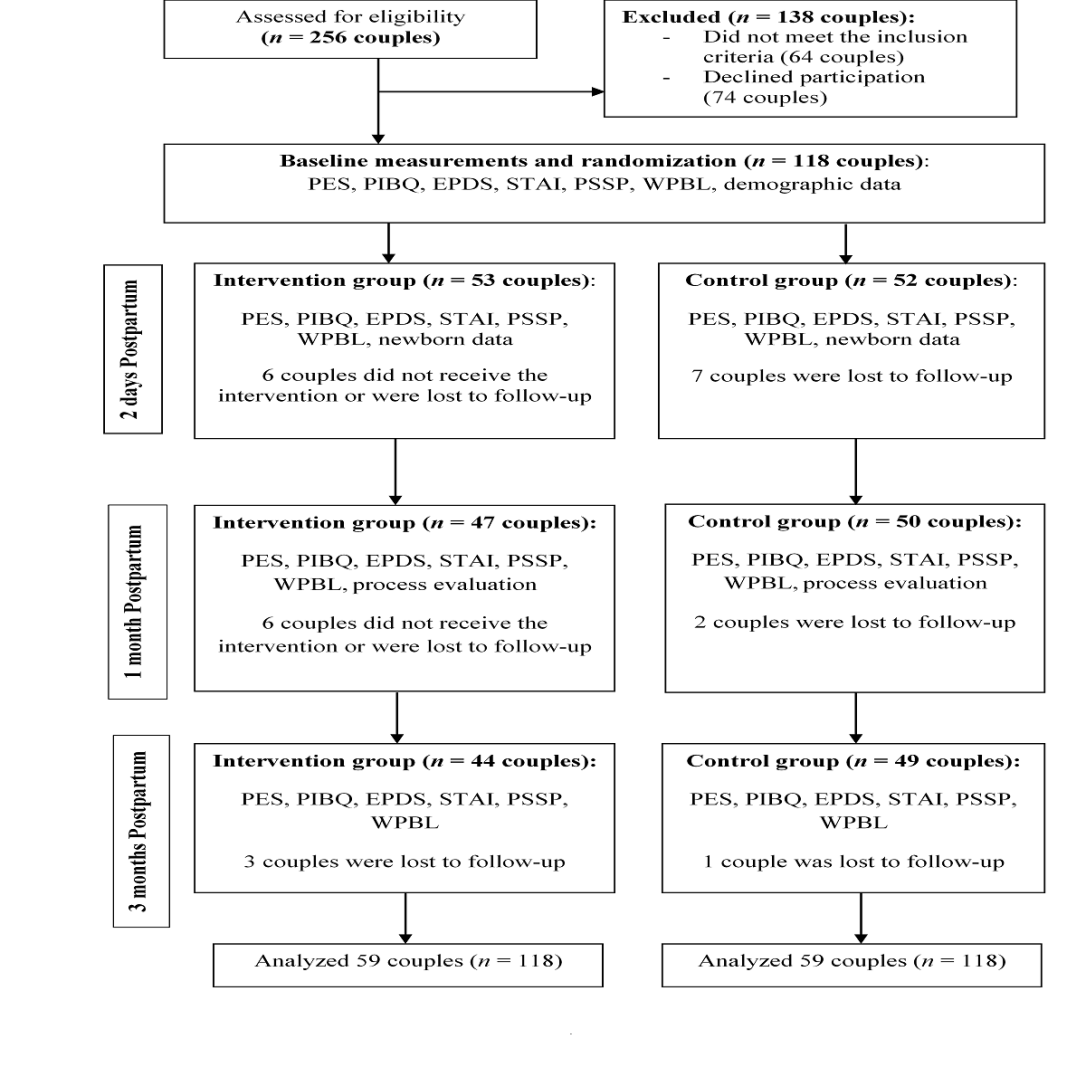
Consolidated standards of reporting trial flowchart of the study. EPDS: Edinburgh Postnatal Depression Scale; PES: Parenting Efficacy Scale; PIBQ: Parent-to-Infant Bonding Questionnaire; PSSP: Perceived Social Support for Parenting; STAI: State Trait Anxiety Inventory; WPBL: What Being a Parent of a Baby Is Like.

A total of 236 (118 couples) participants were recruited and randomized into the SEPP intervention group (n=59) and control group (n=59). The baseline sociodemographic and pregnancy- related characteristics of the study participants are presented in Table 1. Participants had a mean age of 32 years (SD 4.81, range 22-51). All participants were married with an average marriage length of 3.5 years (SD 2.67, range 1-10). The majority of the participants were Chinese (109/236, 46.2%), university graduates (173/236, 73.3%), and employed (215/236, 91.1%) with a household income of more than SGD $5000 (138/236, 58.5%). The majority of the participants did not attend antenatal classes; most mothers had a normal vaginal delivery and followed a confinement period. There were no statistically significant differences between the control and intervention groups on demographic characteristics except for age (*P*=.026) and length of marriage (*P*=.008). Figure 1 shows the Consolidated Standards of Reporting Trial flowchart of the study.

**Table 1:** Couples (mothers and fathers) population: summary of sociodemographic and pregnancy-related characteristics at the baseline.

Characteristics		Intervention (n=118)	Control (n=118)
**Age (years), mean (SD; min, max)**			
	Couples	31.3 (4.6; 22, 51)	32.6 (5.0; 24, 51)
	Fathers	32.1 (4.6; 25, 51)	33.9 (5.1; 26, 51)
	Mothers	30.4 (4.4; 22, 42)	31.4 (4.6; 24, 45)
**Ethnicity, n (%)**			
	Chinese	54 (45.8)	55 (46.6)
	Malay	28 (23.7)	32 (27.1)
	Indian	22 (18.6)	17 (14.4)
	Others	14 (11.9)	14 (11.9)
Marriage length (years), mean (SD; min, max)		3.1 (2.5; 1, 10)	4.0 (2.8; 1, 10)
**Educational level, n (%)**			
	Primary/secondary school	7 (5.9)	1 (0.9)
	Diploma/polytechnic	28 (23.7)	26 (22.2)
	University graduates	83 (70.3)	90 (76.3)
Employed participants, n (%)		106 (89.8)	110 (93.2)
**Monthly household income, n (%)**			
	<SGD $3000	18 (15.3)	12 (10.2)
	SGD $3000-$5000	36 (30.5)	30 (25.4)
	>SGD $5000	62 (52.5)	76 (64.4)
Planned pregnancy, n (%)		80 (67.8)	80 (67.8)
Attended antenatal class, n (%)		40 (33.9)	31 (26.3)
**Mode of delivery, n (%)**			
	Normal vaginal delivery/water birth	72 (61.0)	76 (64.4)
	Instrumental delivery	8 (6.8)	4 (3.4)
	Cesarean section	32 (27.1)	32 (27.1)
Female babies, n (%)		48 (40.7)	48 (40.7)
**Birth order, n (%)**			
	First	84 (71.2)	74 (62.7)
	Second	22 (18.6)	36 (30.5)
	Third and above	10 (8.5)	4 (3.4)
**Paternal/maternal leave, n (%)**			
	No leave	3 (2.5)	2 (1.7)
	≤12 weeks	69 (58.5)	61 (51.7)
	>12 weeks	30 (25.4)	40 (33.9)
Confinement period, n (%)		98 (83.1)	94 (79.7)
**Mode of feeding, n (%)**			
	Breastfeeding	74 (62.7)	70 (59.3)
	Formula feeding	2 (1.7)	4 (3.4)
	Breastfeeding and formula feeding	34 (28.8)	42 (35.6)

Follow-up assessments at 1 month postpartum were completed for 47 couples (47/59, 80%) in the intervention group and 50 couples (50/59, 85%) in the control group. At 3 months postpartum, follow-up assessments were completed for 44 couples (44/59, 75%) in the intervention group and 49 couples (49/59, 83%) in the control group. However, as the intention-to-treat analysis was adopted, data were analyzed for all 59 couples in both control and intervention groups. The overall attrition rate was 21.8%. Outcome scores were adjusted for ethnicity, maternal/paternal leave, confinement period, infant feeding mode, age, length of marriage, household income, employment status, and education. Table 2 summarizes the mean scores for parental outcomes of PSE, parental bonding, PND, PNA, perceived social support, and parenting satisfaction in the intervention and control groups at each time point. Multimedia Appendix 1 shows key baseline characteristics of participants who provided all data (complete case analysis) and those who provided partial data. As compared with participants who provided complete data, participants excluded from the complete case analysis were married slightly longer, had lower education, had less monthly household income, and fewer of them practiced the confinement period; therefore, the results of the unadjusted complete case analysis may or may not be extrapolated to this group of parents.

**Table 2:** Couples (mothers and fathers) population: summary of parental outcomes in the control (n=118) and intervention groups (n=118) at baseline, 2 days, 1 month, and 3 months postpartum.

Outcomes	Baseline, mean (SD)		2 days postpartum, mean (SD)		1 month postpartum, mean (SD)		3 months postpartum, mean (SD)	
	Control	Intervention	Control	Intervention	Control	Intervention	Control	Intervention
Parenting self-efficacy (10-40)	28.45 (6.2)	29.91 (5.8)	27.37 (5.9)	27.27 (6.3)	29.46 (6.2)	29.07 (5.9)	32.10 (4.8)	31.95 (4.4)
Parental bonding (PIBQ^a^, 0-21)	2.73 (3.1)	2.54 (3.1)	1.84 (2.2)	1.58 (1.6)	14.05 (2.8)	1.30 (1.7)	5.64 (6.0)	1.37 (1.7)
Postnatal depression (EPDS^b^, 0-30)	6.02 (4.1)	6.39 (4.1)	5.96 (4.6)	6.09 (3.8)	4.76 (4.9)	5.65 (4.2)	4.16 (4.3)	5.37 (4.1)
Postnatal anxiety (STAI^c^, 40-160)	67.46 (17.2)	68.23 (17.8)	66.47 (19.2)	69.29 (18.1)	64.34 (21.9)	66.97 (17.8)	61.28 (16.7)	62.29 (18.2)
Perceived social support (PSSP^d^, 0-40)	33.38 (5.8)	32.14 (7.5)	35.86 (4.4)	35.41 (7.7)	34.53 (6.0)	33.27 (6.5)	35.72 (4.3)	33.36 (7.2)
Parenting satisfaction (WPBL^e^, 0-99)	85.32 (12.2)	82.14 (12.1)	83.32 (12.1)	81.18 (12.7)	83.87 (13.5)	82.8 (12.2)	88.85 (8.7)	87.61 (10.5)

^a^PIBQ: Parent-to-Infant Bonding Questionnaire.

^b^EPDS: Edinburgh Postnatal Depression Scale.

^c^STAI: State Trait Anxiety Inventory.

^d^PSSP: Perceived Social Support for Parenting.

^e^WPBL: What Being a Parent of a Baby Is Like.

### Main Analysis

**Figure 2:**
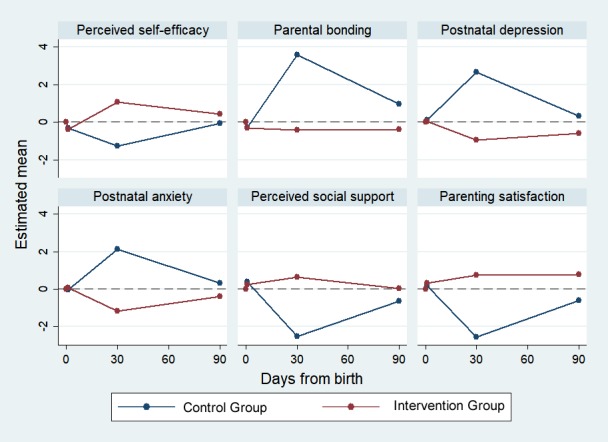
Couples’ (mothers and fathers) population: changes in the standardized estimated mean scores of parental outcomes at 2 days, 1 month, and 3 months postpartum in control and intervention groups.

The mean difference of the standardized scores between the control and intervention groups immediately postpartum from the baseline (ie, intervention × time effects) was not significant for all parental outcomes (PSE, parental bonding, PND, PNA, social support, and parenting satisfaction; Table 3). At 1 month postpartum, the mean difference of the standardized scores between the control and intervention groups from the baseline was significant for all parental outcomes except PNA (difference [*d*]=−3.30; 95% CI −8.17 to 1.57; *P*=.07). However, after adjusting for covariates, the mean difference of the standardized score between the groups for PNA was significant (*d*=−3.25; 95% CI −3.65 to −2.85; *P*<.001). At 3 months postpartum, all mean difference scores between the groups were significant for all parental outcomes. The mean difference between the 2 groups at the baseline (ie, main effect of intervention) was close to 0 for all outcomes (each *P*>.05; results not shown). A comparison of the mean outcome scores between the control and intervention groups across all time points is shown in Figure 2. Sensitivity analyses based on the complete case and multiple imputed data showed results similar to the main analysis (Multimedia Appendices 2 and 3).

### Subgroup Analysis

For the analysis of parental outcome scores for mothers, the mean difference of scores between groups immediately postpartum was not significant for all outcomes, but the mean difference of scores between groups (ie, intervention × time effects) was significant for all parental outcomes at 1 month postpartum. At 3 months postpartum, the mean difference of the scores was only significant for parental bonding (*d*=−1.33; 95% CI −1.92 to −0.74; *P*<.001), PND (*d*=−0.84; 95% CI −1.24 to −0.44; *P*<.001), PNA (*d*=−0.55; 95% CI −0.93 to −0.17; *P*=.01), and parenting satisfaction (*d*=1.31; 95% CI 0.72 to 1.90; *P*<.001). After adjusting for covariates, the mean difference of the scores for social support became significant (*d*=0.69; 95% CI 0.06 to 1.33; *P*=.03), whereas the mean difference of the scores for PSE remained as not significant (*d*=0.33; 95% CI −0.20 to 0.85; *P*=.22).

Similarly, for the analysis of parental outcome scores for fathers, the mean difference of the scores between the groups was not significant immediately postpartum. However, the mean difference of the scores for all parental outcomes was significant at 1 and 3 months postpartum. The mean difference between the 2 groups at the baseline (ie, main effect of intervention) was close to 0 for all outcomes (each *P*>.05) in models for mothers and fathers (results not shown). A summary of the mean difference of standardized parental outcome scores between the groups for mothers and fathers is shown in Tables 4 and 5, respectively.

**Table 3:** Couple (mothers and fathers) population: estimated differences between the intervention and control groups for changes in standardized parental outcomes at 2 days, 1 month, and 3 months postpartum.

Standardized outcomes	2 days postpartum				1 month postpartum				3 months postpartum			
	Unadjusted difference^a^		Adjusted difference^b^		Unadjusted difference		Adjusted difference		Unadjusted difference		Adjusted difference	
	OR^c^ (95% CI)	*P* value	OR (95% CI)	*P* value	OR (95% CI)	*P* value	OR (95% CI)	*P* value	OR (95% CI)	*P* value	OR (95% CI)	*P* value
Parenting self-efficacy	−0.20 (−0.53 to 0.12)	0.21	−0.29 (−0.75 to 0.17)	0.21	2.23 (1.92 to 2.54)	<.001	2.36 (1.94 to 2.79)	<.001	0.37 (0.06 to 0.68)	0.021	0.45 (0.03 to 0.86)	0.034
Parental bonding	−0.03 (−0.36 to 0.31)	0.88	−0.08 (−0.47 to 0.31)	0.67	−4.00 (−4.39 to −3.60)	<.001	−4.07 (−4.50 to −3.64)	0.001	−1.32 (−1.89 to −0.75)	<.001	−1.53 (−2.17 to −0.89)	<.001
Postnatal depression	−0.05 (−0.33 to 0.22)	0.69	0.05 (−0.26 to 0.35)	0.76	−3.40 (−3.97 to −3.22)	<.001	−3.54 (−3.93 to −3.15)	<.001	−0.91 (−1.34 to −0.49)	<.001	−0.92 (−1.38 to −0.47)	<.001
Postnatal anxiety	0.11 (−0.14 to 0.36)	0.37	0.15 (−0.12 to 0.43)	0.26	−3.30 (−8.17 to 1.57)	0.07	−3.25 (−3.65 to −2.85)	<.001	−0.71 (−1.01 to −0.42)	<.001	−0.82 (−1.15 to −0.49)	<.001
Perceived social support	−0.13 (−0.42 to 0.16)	0.37	−0.18 (−0.47 to 0.10)	0.19	3.14 (2.75 to 3.53)	<.001	3.13 (2.78 to 3.47)	<.001	0.69 (0.18 to 1.19)	0.008	0.76 (0.36 to 1.16)	<.001
Parenting Satisfaction	0.14 (−0.21 to 0.49)	0.44	0.03 (−0.37 to 0.34)	0.83	3.31 (2.92 to 3.69)	<.001	3.48 (3.14 to 3.83)	<.001	1.40 (0.86 to 1.93)	<.001	1.44 (1.05 to 1.82)	<.001

^a^Unadjusted differences were estimated using a linear mixed model adjusted for baseline values.

^b^Adjusted differences were estimated using the same model with additions of covariates: ethnicity, maternal/paternal leave, confinement period, infant feeding mode, age, length of marriage, household income, employment status, and education. See Methods for outcome definitions.

^c^OR: odds ratio.

**Table 4:** Mothers-only subgroup: estimated differences between intervention and control groups for changes in standardized parental outcomes at immediately, 1 month, and 3 months postpartum.

Standardized outcomes	Immediately postpartum				1 month postpartum				3 months postpartum			
	Unadjusted difference^a^		Adjusted difference^b^		Unadjusted difference		Adjusted difference		Unadjusted difference		Adjusted difference	
	OR^c^ (95% CI)	*P* value	OR (95% CI)	*P* value	OR (95% CI)	*P* value	OR (95% CI)	*P* value	OR (95% CI)	*P* value	OR (95% CI)	*P* value
Parenting self-efficacy	−0.12 (−0.49 to 0.26)	.54	−0.22 (−0.76 to 0.31)	.41	2.29 (1.92 to 2.67)	<.001	2.38 (1.89 to 2.88)	<.001	0.29 (−0.09 to 0.68)	.13	0.33 (−0.20 to 0.85)	.22
Parental bonding	−0.04 (−0.41 to 0.33)	.82	−0.19 (−0.62 to 0.25)	.39	−3.98 (−4.44 to −3.51)	<.001	−4.25 (−4.79 to −3.70)	<.001	−1.33 (−1.92 to −0.74)	<.001	−1.50 (−2.26 to −0.74)	<.001
Postnatal depression	−0.33 (−0.68 to 0.03)	.07	−0.15 (−0.54 to 0.25)	.46	−3.69 (−4.08 to −3.30)	<.001	−3.57 (−4.00 to −3.15)	<.001	−0.84 (−1.24 to −0.44)	<.001	−0.81 (−1.25 to −0.37)	<.001
Postnatal anxiety	−0.02 (−0.39 to 0.35)	.91	0.17 (−0.22 to 0.57)	.39	−3.26 (−3.72 to −2.79)	<.001	−3.32 (−3.80 to −2.84)	<.001	−0.55 (−0.93 to −0.17)	.005	−0.55 (−0.94 to −0.15)	.007
Perceived social support	−0.03 (−0.40 to 0.33)	.85	−0.10 (−0.52 to 0.33)	.65	3.36 (2.85 to 3.86)	<.001	3.40 (2.86 to 3.95)	<.001	0.57 (−0.06 to 1.20)	.075	0.69 (0.06 to 1.33)	.033
Parenting satisfaction	0.02 (−0.42 to 0.46)	.92	−0.15 (−0.61 to 0.32)	.53	3.64 (2.98 to 4.30)	<.001	3.77 (2.92 to 4.63)	<.001	1.31 (0.72 to 1.90)	<.001	1.31 (0.61 to 2.02)	<.001

^a^Unadjusted differences were estimated using a linear mixed model adjusted for baseline values.

^b^Adjusted differences were estimated using the same model with additions of covariates: ethnicity, maternal/paternal leave, confinement period, infant feeding mode, age, length of marriage, household income, employment status, and education. See Methods for outcome definitions.

^c^OR: odds ratio.

**Table 5:** Fathers-only subgroup: estimated differences between the intervention and control groups for changes in standardized parental outcomes at immediately, 1 month, and 3 months postpartum.

Standardized outcomes	Immediately postpartum				1 month postpartum				3 months postpartum			
	Unadjusted difference^a^		Adjusted difference^b^		Unadjusted difference		Adjusted difference		Unadjusted difference		Adjusted difference	
	OR^c^ (95% CI)	*P* value	OR (95% CI)	*P* value	OR (95% CI)	*P* value	OR (95% CI)	*P* value	OR (95% CI)	*P* value	OR (95% CI)	*P* value
Parenting self-efficacy	−0.28 (−0.77 to 0.21)	.26	−0.40 (−0.91 to 0.11)	.12	2.15 (1.64 to 2.66)	<.001	2.13 (1.59 to 2.68)	<.001	0.48 (0.01 to 0.95)	.044	0.51 (0.03 to 0.99)	.036
Parental bonding	0.01 (−0.37 to 0.39)	.94	−0.04 (−0.49 to 0.41)	.84	−3.99 (−4.40 to −3.59)	<.001	−4.31 (−4.78 to −3.84)	<.001	−1.29 (−1.80 to −0.77)	<.001	−1.48 (−2.22 to −0.75)	<.001
Postnatal depression	0.11 (−0.24 to 0.45)	.53	0.13 (−0.23 to 0.49)	.46	−3.56 (−3.94 to −3.17)	<.001	−3.58 (−4.07 to −3.10)	<.001	−0.92 (−1.33 to −0.50)	<.001	−1.09 (−1.68 to −0.49)	<.001
Postnatal anxiety	0.17 (−0.14 to 0.48)	.26	0.13 (−0.18 to 0.44)	.4	−3.37 (−3.74 to −3.00)	<.001	−3.40 (−3.93 to −2.86)	<.001	−0.90 (−1.26 to −0.54)	<.001	−1.09 (−1.57 to −0.61)	<.001
Perceived social support	−0.22 (−0.57 to 0.13)	.22	−0.22 (−0.62 to 0.18)	.28	3.00 (2.61 to 3.39)	<.001	2.98 (2.52 to 3.44)	<.001	0.67 (0.18 to 1.15)	.007	0.81 (0.16 to 1.45)	.015
Parenting satisfaction	0.26 (−0.09 to 0.61)	.14	0.21 (−0.23 to 0.64)	.35	3.45 (3.08 to 3.81)	<.001	3.45 (3.01 to 3.89)	<.001	1.45 (1.02 to 1.88)	<.001	1.55 (0.95 to 2.16)	<.001

^a^Unadjusted differences were estimated using a linear mixed model adjusted for baseline values.

^b^Adjusted differences were estimated using the same model with additions of covariates: ethnicity, maternal/paternal leave, confinement period, infant feeding mode, age, length of marriage, household income, employment status, and education. See Methods for outcome definitions.

^c^OR: odds ratio.

## Discussion

### Overview

This study examined the effectiveness of a technology-based SEPP among parents in Singapore. As the first 2 days postpartum is a tumultuous time for parents, those in the intervention group did not show enhanced parental outcomes during the immediate postpartum period. However, they had significantly better parental outcome scores than those in the control group at 1 and 3 months postpartum, which suggests the effectiveness of the intervention. In terms of score trends for parental outcomes between 1 and 3 months postpartum, there are noticeable increases in the outcome scores for PSE, social support, and parenting satisfaction and decreases in the outcome scores for PND, parental bonding, and PNA for the control group. For the intervention group, scores for PSE, social support, and parenting satisfaction decreased, whereas scores for PND, PNA, and parental bonding increased. This implied that the cessation of the mHealth app usage at 1 month caused a decrease in parental outcomes.

### Parenting Self-Efficacy

The significantly better PSE scores for the intervention group suggest that the SEPP is effective in enhancing parents’ confidence in infant care skills and capability as a parent. Adhering to Bandura’s self-efficacy theory [12], components of this technology-based SEPP allowed parents to obtain self-efficacy through mastery experiences (via telephone-based educational sessions and mobile phone app content information), vicarious experiences (via learning from other parents on the forum), and verbal persuasion (via constructive feedback and encouragement from the midwife).

The positive results for parental PSE in the intervention group were similar to a Finnish study by Salonen et al where parents who received a Web-based educational intervention reported significant increases in PSE during the postpartum period [30]. However, another study by Bartholomew et al that evaluated the frequency of Facebook usage and communication with Facebook friends by parents during the postpartum period found no significant increase in PSE [55]. This suggests that apart from social support, educational information is essential in enhancing PSE among parents. The effectiveness of the technology-based SEPP in boosting PSE in fathers and mothers also corresponds with other parent-specific Web-based intervention studies (eg, Home-but-not-alone [31], Mom Mood Booster [56], and New Fathers Network [28]), which showed significantly better PSE scores in the intervention group around 1 to 6 months postpartum.

After the cessation of the mHealth app usage, the PSE of parents in the intervention group decreased slightly, whereas PSE in the control group increased, resulting in similar scores. This suggested that parents in the control group needed 3 months of mastery experience to reach the same level of PSE as parents in the intervention group, who were able to achieve high levels of PSE within 1 month. These results corresponded with Porter and Hsu’s study on first-time mothers, which reported a significant increase in maternal self-efficacy at 3 months postpartum even without an educational intervention [57]. A pilot study in Iran discovered an improvement in maternal self-efficacy after 6 weeks of an intervention and no change in PSE even 1 month after the withdrawal of the intervention [58]. Along with our results, this supports Bandura’s theory [12] that childcare experience increases maternal PSE regardless of the intervention received and that the SEPP is only effective in helping parents gain PSE in a shorter time.

### Parental Bonding

There were significant differences in parental bonding scores between the control and intervention groups at 1 month and 3 months postpartum. According to Bowlby’s attachment theory, good parental bonding is dependent on high PSE, good psychological health, and good social support [16]. This corresponds with a recent review by Edward et al who reported PND and poor maternal social networks as predictors of impaired maternal bonding [49]. As the SEPP was effective in increasing PSE, parents were more confident in infant care skills such as breastfeeding, bathing, and swaddling the baby. These interactions and increased skin-to-skin contact [59] helped to release the oxytocin hormone, which is essential for parent-infant bonding [60].

Results from existing studies on the effectiveness of educational interventions on parental bonding are still inconclusive. Some studies have found increased parental bonding after receiving an intervention [61-63], whereas other studies reported no change in parental bonding [53,54,64]. Parental bonding plays an important role in promoting the healthy psychosocial well-being of parents and social development of the child [61,63]; therefore, more rigorous testing is required to determine the effectiveness of such educational interventions on parental bonding.

### Social Support

The intervention group scored significantly higher than the control group for perceived social support at 1 month postpartum. This implied that the rich knowledge-based content and video and audio recordings of infant care were effective in providing professional informational support to both parents. The online discussion forum provided emotional support by creating a social support system among parents that fostered a sense of belonging within the community. This result corresponded with a local study, which found that the use of an informational mobile app significantly improved perceived social support of mothers during the postpartum period [31]. Two reviews further revealed that such online-based forums are common sources of peer support, helping to reduce stigma and promoting help-seeking behavior, which reduces PND and PNA [9,65]. An increasing number of young parents are also turning to the internet for social support rather than to their family and friends [10]. A combination of professional and peer support was also found to increase the efficacy of educational programs [66].

On the contrary, the decrease in scores for both groups at 3 months postpartum could be attributed to the cessation of the mHealth app usage (for the intervention group), which removed an essential source of social support, or it could be due to other external factors such as the intrusiveness of family members/in-laws [67-70] or the 1-month confinement period itself [4,5]. However, further studies are required to validate these findings.

### Postnatal Depression and Postnatal Anxiety

For PND, there were significant differences between the control and intervention groups at 1 month and 3 months postpartum. However, in the intervention group, PND scores increased after the termination of SEPP, indicating more depressive symptoms. This suggested that the SEPP is effective in mitigating PND and the lack of it increases the risk of PND. These results were contrary to a local study where an educational mHealth app administered for 1 month postpartum did not show significant results in mitigating PND at 3 months postpartum [31]. However, a study by Danaher et al involving a 6- to 12-week-long Web-based intervention for PND prevention among mothers found significant decrease in PND scores at 3 and 6 months postpartum [56]. Despite a similar outcome to the present results, more research is required to determine the effects of length of intervention and follow-up time points on the effectiveness of technology-based psychoeducational programs on PND prevention.

For PNA also, there were significant differences between intervention and control groups at 1 and 3 months postpartum. A study by Seymour et al reported that postpartum maternal anxiety was associated with poor partner relationship quality, need for social support, low involvement, low efficacy, and low parenting satisfaction [26]. However, comorbid depression and anxiety were more strongly correlated with these negative experiences than anxiety alone [26]. Studies often investigate PNA and depression together because of their high comorbidity rates [71]. According to a study in the Netherlands, in 57% of the comorbid cases, anxiety preceded depression, and in 18% of cases, depression preceded anxiety [72]. In this study, PND and PNA showed similar trends in both groups, with PNA scores being slightly lower than PND at all time points. Similarly, O’Mahen et al reported a significant decline in both PND and PNA scores for mothers at 3 months postpartum using an 11-week technology-based intervention involving weekly phone calls from coaches and Web-based information [73]. Although few studies focused on the comorbidity of depression and anxiety in fathers [74], previous studies have mentioned the spillover effects of maternal psychological health on paternal psychological health [75], which can adversely affect the development of the child [74]. However, coparenting alliance and paternal self-efficacy were found to reduce the paternal stress and overall psychological health of fathers [76,77]. Therefore, couple-based educational programs are important for facilitating partner support, ensuring each parent’s well-being, and increasing parenting dynamics [78-80].

### Parenting Satisfaction

There was an increase in parenting satisfaction scores in the intervention group, whereas there was a large decrease in parenting satisfaction scores in the control group. These results were congruent with Salonen et al’s study in which mothers who received a Web-based educational intervention reported higher parenting satisfaction, but not fathers [30]. However, Hudson et al reported an increase in paternal parenting satisfaction after an online fathers’ network intervention [28]. In both studies, PSE was found to be positively correlated with parenting satisfaction as parents gained more confidence in caring for their baby [28,30]. This suggests that higher PSE scores predict higher levels of parenting satisfaction, which corresponds to our findings.

### Strengths and Limitations

Although most studies focused on technology-based interventions in the postpartum period [28,30,31], this study administered its intervention during the perinatal period, which may increase the effectiveness of improving parental outcomes. This study adds on to existing literature on maternal well-being and fills knowledge gaps on paternal well-being during the perinatal period. Given that most parents today are turning to the internet for information and social support, the technology-based SEPP is an ideal time- and cost-effective method to meet parental needs and reduce dependency on midwives who may not be available.

A major limitation of this study is that as the SEPP is a technology-based intervention, there is no practical hands-on skill incorporated into the program. This reduces the effectiveness of parenting skill-based learning, especially for experiential/hands-on learners. However, the short telephone- based educational classes (30 min and 1 hour) in the SEPP may suit working parents who lack the time to attend antenatal classes, which may last up to 16 hours. Therefore, future studies should investigate the difference in effectiveness between technology-based classes and traditional face-to-face didactic perinatal classes.

Owing to a lack of available data, we were unable to analyze the cost-effectiveness of this program; future research may consider evaluating cost-effectiveness as an outcome. This study lacks infant outcome data, thus restricting the evaluation of actual benefits of intervention on infants. Moreover, this is a single-site study that only included married English-speaking couples. As we believe, the SEPP will be equally beneficial to parents from the minority group (ie, single parents, other races, etc); future research should be more inclusive and consider a multisite study with a minority population. Future studies should also investigate the long-term effectiveness (beyond 3 months) of the SEPP on parental outcomes.

The internal validity of PIBQ was not optimal to measure parental bonding among multiracial parents in this study, although it has been validated in previous studies [44,81,82]. This could be due to cultural influence on parental bonding and that some questionnaire items may require further evaluation. Nevertheless, the precision of the intervention effect based on the PIBQ was not compromised because of the adequately large sample size of the study. However, we strongly recommend further testing of this instrument among various cultural populations and the testing of its psychometric properties.

### Conclusions

This study demonstrated the effectiveness of a technology-based SEPP in improving PSE, parental bonding, perceived social support, and parenting satisfaction, while reducing PND and PNA. Such educational programs are vital to equip parents with the necessary parenting skills to facilitate a smoother transition into parenthood by increasing their self-efficacy and enhancing parental bonding. They also serve as a reliable source of social support to promote good psychological health in both fathers and mothers. We hope that the clinical implementation of the SEPP will meet parents’ needs and create positive childbirth experiences, which may in turn encourage parents to have more children and alleviate Singapore’s declining birth rate.

## Appendix

Multimedia Appendix 1 Comparison of sociodemographic and pregnancy-related characteristics of participants at the baseline who were included and excluded from the complete case analysis.

Multimedia Appendix 2 Complete cases analysis for couple population (mothers and fathers): estimated differences between the intervention and control groups for changes in standardized parental outcomes at postpartum time-points from the baseline.

Multimedia Appendix 3 Multiple imputation analyses for couple population (mothers and fathers): estimated differences between the intervention and control groups for changes in standardized parental outcomes at postpartum time points from the baseline.

Multimedia Appendix 4 CONSORT‐EHEALTH checklist (V 1.6.1).

## Abbreviations

EPDS: Edinburgh Postnatal Depression Scale
mHealth: mobile health
OR: odds ratio
PES: Parenting Efficacy Scale
PIBQ: Parent-to-Infant Bonding Questionnaire
PNA: postnatal anxiety
PND: postnatal depression
PSE: parenting self-efficacy
PSSP: Perceived Social Support for Parenting
RA: research assistant
SEPP: supportive educational parenting program
STAI: State Trait Anxiety Inventory
WPBL: What Being a Parent of a Baby Is Like
