# Supplementary material for: Effectiveness of a Technology-Based Supportive Educational Parenting Program on Parental Outcomes (Part 1): Randomized Controlled Trial
Source: J Med Internet Res. 2019 Feb 13;21(2):e10816. doi: 10.2196/10816 (PMC6391716; doi:10.2196/10816)
Supplement: Multimedia Appendix 2 [file jmir_v21i2e10816_app2.pdf]

Multimedia Appendix 2. Complete cases analysis for couple population (mothers and fathers): Estimated differences between the intervention and control groups for changes in standardized parental outcomes at postpartum time-points from the baseline

	Immediately postpartum		1 month postpartum		3 months postpartum	
	Unadjusted	Adjusted	Unadjusted	Adjusted	Unadjusted	Adjusted
Standardized outcomes	Difference (95% CI) [*P*]	Difference (95% CI) [*P*]	Difference (95% CI) [*P*]	Difference (95% CI) [*P*]	Difference (95% CI) [*P*]	Difference (95% CI) [*P*]
Parental self-efficacy	-0.11 (-0.47, 0.26) [0.561]	-0.30 (-0.91, 0.30) [0.168]	2.23 (1.88, 2.57) [<0.001]	2.26 (1.97, 2.54) [<0.001]	0.43 (0.08, 0.77) [0.015]	0.43 (0.14, 0.71) [0.003]
Parental bonding	-0.16 (-0.50, 0.18) [0.354]	-0.28 (-0.71, 0.14) [0.189]	-4.12 (-4.48, -3.76) [<0.001]	-4.24 (-4.68, -3.81) [<0.001]	-1.49 (-1.91, -1.06) [<0.001]	-1.77 (-2.35, -1.19) [<0.001]
Postnatal depression	-0.11 (-0.42, 0.19) [0.458]	0.04 (-0.28, 0.36) [0.800]	-3.71 (-1.04, -3.39) [<0.001]	-3.56 (-3.91, -3.21) [<0.001]	-0.94 (-1.28, -0.60) [<0.001]	-0.98 (-1.34, -0.63) [<0.001]
Postnatal anxiety	0.19 (-0.09, 0.47) [0.180]	0.26 (-0.05, 0.56) [0.103]	-3.30 (-3.77, -2.84) [<0.001]	-3.20 (-3.34, -2.77) [<0.001]	-0.76 (-1.11, -0.42) [<0.001]	-0.79 (-1.15, -0.43) [<0.001]
Perceived social support	-0.16 (-0.48, 0.15) [0.311]	-0.27 (-0.63, 0.08) [0.126]	3.09 (2.67, 3.50) [<0.001]	2.95 (2.51, 3.39) [<0.001]	0.71 (0.13, 1.28) [0.017]	0.81 (0.19, 1.43) [0.011]
Parenting satisfaction	0.01 (-0.40, 0.43) [0.944]	-0.04 (-0.45, 0.36) [0.834]	3.30 (2.81, 3.78) [<0.001]	3.25 (2.79, 3.71) [<0.001]	1.45 (0.84, 2.06) [<0.001]	1.48 (0.87, 2.08) [<0.001]

Unadjusted differences were estimated using a linear mixed model adjusted for baseline values. Adjusted differences were estimated using the same model with additions of covariates ethnicity, maternal/paternal leave, confinement period, infant feeding mode, age, length of marriage, household income, employment status, and education. See the Methods section for outcome definitions. CI = Confidence interval.
