# Supplementary material for: Effectiveness of a Technology-Based Supportive Educational Parenting Program on Parental Outcomes (Part 1): Randomized Controlled Trial
Source: J Med Internet Res. 2019 Feb 13;21(2):e10816. doi: 10.2196/10816 (PMC6391716; doi:10.2196/10816)
Supplement: Multimedia Appendix 3 [file jmir_v21i2e10816_app3.pdf]

Multimedia Appendix 3. Multiple imputation analyses for couple population (mothers and fathers): Estimated differences between the intervention and control groups for changes in standardized parental outcomes at postpartum timepoints from the baseline

	Immediately postpartum		1 month postpartum		3 months postpartum	
	Unadjusted	Adjusted	Unadjusted	Adjusted	Unadjusted	Adjusted
Standardized outcomes	Difference (95% CI) [*P*]	Difference (95% CI) [*P*]	Difference (95% CI) [*P*]	Difference (95% CI) [*P*]	Difference (95% CI) [*P*]	Difference (95% CI) [*P*]
Parental self-efficacy	-0.42 (-0.99, 0.15) [0.150]	-0.42 (-0.99, 0.15) [0.152]	1.99 (1.39, 2.59) [<0.001]	1.98 (1.38, 2.58) [<0.001]	1.99 (1.39, 2.59) [<0.001]	1.98 (1.38, 2.58) [<0.001]
Parental bonding	-0.12 (-0.46, 0.21) [0.467]	-0.13 (-0.49, 0.23) [0.479]	-3.50 (-3.94, -3.09) [<0.001]	-3.49 (-3.87, -3.12) [<0.001]	-1.26 (-1.73, -0.79) [<0.001]	-1.25 (-1.63, -0.87) [<0.001]
Postnatal depression	-0.16 (-0.56, 0.24) [0.438]	-0.15 (-0.56, 0.26) [0.465]	-3.64 (-4.13, -3.16) [<0.001]	-3.64 (-4.15, -3.13) [<0.001]	-3.64 (-4.13, -3.16) [<0.001]	-3.64 (-4.15, -3.13) [<0.001]
Postnatal anxiety	0.03 (-0.34, 0.39) [0.881]	0.06 (-0.57, 0.69) [0.849]	-3.32 (-3.81, -2.82) [<0.001]	-3.25 (-4.34, -2.16) [<0.001]	-3.32 (-3.81, -2.82) [<0.001]	-3.30 (-3.70, -2.91) [<0.001]
Perceived social support	-0.22 (-1.18, 0.75) [0.657]	-0.17 (-0.62, 0.29) [0.474]	3.05 (2.00, 4.10) [<0.001]	3.10 (2.56, 3.64) [<0.001]	3.05 (2.00, 4.10) [<0.001]	3.10 (2.56, 3.64) [<0.001]
Parenting satisfaction	0.38 (-0.11, 0.86) [0.128]	0.377 (-0.12, 0.87) [0.134]	3.78 (3.19, 4.36) [<0.001]	3.77 (3.18, 4.37) [<0.001]	3.77 (3.19, 4.6) [<0.001]	3.77 (3.18, 4.37) [<0.001]

Unadjusted differences were estimated using a linear mixed model adjusted for baseline values. Adjusted differences were estimated using the same model with additions of covariates ethnicity, maternal/paternal leave, confinement period, infant feeding mode, age, length of marriage, household income, employment status, and education. See the Methods section for outcome definitions. CI = Confidence interval.
