# Supplementary material for: Effectiveness of a Technology-Based Supportive Educational Parenting Program on Parental Outcomes (Part 1): Randomized Controlled Trial
Source: J Med Internet Res. 2019 Feb 13;21(2):e10816. doi: 10.2196/10816 (PMC6391716; doi:10.2196/10816)
Supplement: Multimedia Appendix 1 [file jmir_v21i2e10816_app1.pdf]

Dear Editor-in-Chief,

Thank you very much for the opportunity to revise this manuscript again. The necessary amendments have been made as per yours and the reviewer's kind suggestions. Please find point-by-point response to reviewer's comments in the table below.

**Reviewer F's Comments**	**Responses**
General Comments:<br>=================<br>The authors have accommodated reviewer comments. However, responding to reviewer comments has also led to a couple of issues that need to be solved. Most important, the PIBQ results. In addition, the manuscript should be grammar and spell-checked by a native English-speaking person.	Thank you very much for your additional comments on PIBQ results. The necessary amendments have been made to address your concerns, and the manuscript has been re-reviewed for language editing by the Medical Publications Support Unit of the National University Health System, Singapore.
Specific comments<br>================<br>ABSTRACT<br>1. Should it not be 6 months, rather than 12 months: "Data were collected over 12 months at four timepoints: during pregnancy (third trimester), two-days postpartum, one month postpartum, and three months postpartum"?	We apologize for the oversight, yes it should have been 6 months. We have since corrected the error in the Abstract on Page 3.
METHODS<br>2. The Cronbach alpha coefficient for the PIBQ was low and cannot be considered acceptable. Unless I have missed something, you have not taken any measures to problem-solve this. This draws the PIBQ results into question and cannot currently be considered publishable. For readers it is a question of what to make of the PIBQ results and whether these are reliable? Although the Cronbach alpha has been much debated over the years, and can be used to undermine its' importance, it can still be of great practical value, questioning some of its psychometric properties. If you consider it important to present the PIBQ results, you need to improve its' properties either by 1), if	Thank you very much once again for your comments with potential suggestions. We truly appreciate the critical analysis.<br><br>We performed an exploratory factor analysis (EFA) as suggested for the PIBQ data, separately for each visit.<br>EFA showed 2 to 3 factor solutions for different visits, with Eigen values greater than 1. We rerun the EFA with a randomly selected 50% of participants and it showed 3 to 4 factor solutions for different visit data. These indicate that it might not be possible to evaluate whether indeed the PIBQ is multi-dimensional or that multiple factors are an artifact effect of smaller sample size.

possible, adding or replacing items that are currently in use, but which measure the same concept, 2) removing items one-by-one that you suspect may be causing low alphas (this can easily be checked in SPSS, but also consider if there are any items participants may have found difficult to understand or answer), and, either way, 3) perform an exploratory factor analysis (EFA) with maximum likelihood extraction and oblique rotation (i.e., correlated factors). EFA can tell you whether the PIBQ scale is multi-dimensional and should be treated and analyzed as such. I would recommend starting with the EFA on the original items before adding, replacing or removing any items to examine if the problem is lack of uni-dimensionality, in the first place. NB! Cronbach alpha does not tell anything about dimensionality, but low alpha value can be caused by multi-dimensionality (not always, though).	Usually, EFA requires a much larger sample size than we had in the study.<br>Nevertheless, as a next step to improve internal consistency, we evaluated inter-item correlations and found that the 8th item related “Aggressive” bonding was not well correlated with other items. This item focused on how parents bond with their child, especially the feelings they have for their newborn. Showing aggression towards newborns has multi-factorial reasons such as infant factors, family dynamics and parental characteristics etc. [1]<br><br>Based on the Asian family context [2], average household income level, high levels of parental self-efficacy and the satisfaction of parenting by parents in this study, we assumed feelings of aggression by parents towards their newborns as most unlikely. Therefore, we were not surprised that there was no correlation between the 8th item and other items like loving and joyful.<br><br>As such, removal of this item has improved Cronbach alpha values from 0.635, 0.482, 0.568 and 0.570 to 0.704, 0.582, 0.663 and 0.624 for pre-birth, day 2, month 1, and month 3 data respectively. We also adopted Spearman-Brown corrected formula for Cronbach alpha to minimize the impact of less number of items on alpha values.<br><br>We understand that the revised alpha values may not be optimal considering conventional thumb rule of 0.6 or 0.7. We have highlighted this point as a limitation in the discussion section and encouraged further research on improving the scale on Page 28-29.<br><br>All the analyses for PIBQ were re-analyzed using the total score based on 7 items. The revised results have an intervention effect similar to previous ones at each of the

	individual visits.
RESULTS<br>3. Please report the missing data analyses where you compared key baseline characteristics of couples with complete data versus those with missing data such that readers can assess these by themselves. Comparing results with complete case data versus multiple imputation data are not missing data analyses per se and do not tell us anything about potential missing data mechanisms.	We have added Appendix 1 with comparison of key baseline characteristics of couples with complete data versus those with missing data.

1. Reebye P. Aggression during early years - infancy and preschool. Can Child Adolesc Psychiatr Rev. 2005 Feb;14(1):16-20.

2. Md-Yunus, SA. Childcare practices in three Asian countries. Faculty Research and Creative Activity 2005; 10. https://thekeep.eiu.edu/eemedu_fac/10
